# Self-Rectifying Resistive Switching Memory with Ultralow Switching Current in Pt/Ta_2_O_5_/HfO_2-*x*_/Hf Stack

**DOI:** 10.1186/s11671-017-1905-3

**Published:** 2017-02-15

**Authors:** Haili Ma, Jie Feng, Hangbing Lv, Tian Gao, Xiaoxin Xu, Qing Luo, Tiancheng Gong, Peng Yuan

**Affiliations:** 1 Department of Micro/Nano Electronics, Key Laboratory for Thin Film and Micro Fabrication of Ministry of Education, Shanghai Jiao Tong University, Shanghai, China; 2 Key Laboratory of Microelectronics Devices and Integrated Technology, Institute of Microelectronics, Chinese Academy of Sciences, Beijing, China

**Keywords:** RRAM, Crossbar, Leakage current issue, Self-rectifying

## Abstract

In this study, we present a bilayer resistive switching memory device with Pt/Ta_2_O_5_/HfO_2-*x*_/Hf structure, which shows sub-1 μA ultralow operating current, median switching voltage, adequate ON/OFF ratio, and simultaneously containing excellent self-rectifying characteristics. The control sample with single HfO_2-*x*_ structure shows bidirectional memory switching properties with symmetrical I–V curve in low resistance state. After introducing a 28-nm-thick Ta_2_O_5_ layer on HfO_2-*x*_ layer, self-rectifying phenomena appeared, with a maximum self-rectifying ratio (RR) of ~4 × 10^3^ observed at ±0.5 V. Apart from being a series resistance for the cell, the Ta_2_O_5_ rectifying layer also served as an oxygen reservoir which remains intact during the whole switching cycle.

## Background

**Fig. 1:**
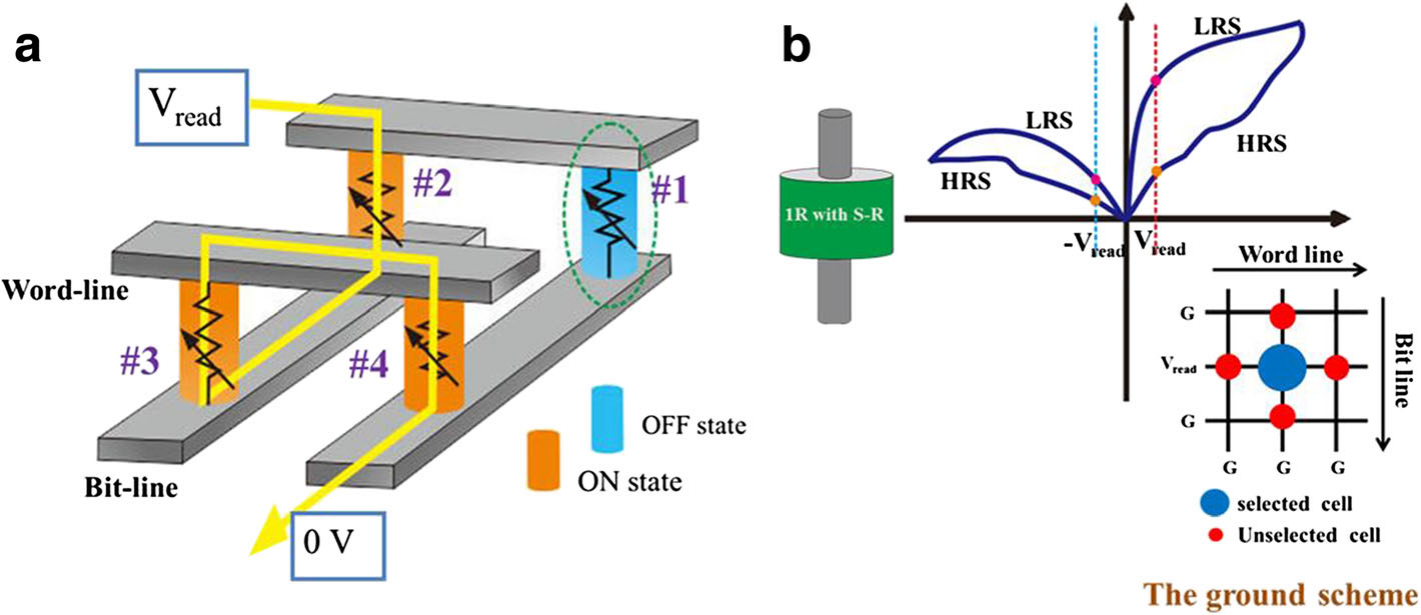
**a** The sneak-path issue happened in crossbar structure and **b** typical I–V characteristics and read scheme of 1R with S-R cells

The long time issue faced by semiconductor industry was searching for efficient methods to overcome the fundamental scaling limits of charge-based information storage devices. One of the promising methods is to use crossbar architecture to increase integration density. However, the sneak-path issue (shown in Fig. 1a) acts as a disturbing problem in reading the correct information [1]. Various methods are developed to settle this urgent problem, including one MOSFET transistor-one resistor (1T1R), one diode-one resistor (1D1R), complementary resistive switch (CRS), one bidirectional selector-one resistor (1S1R), and one resistor with self-rectification (1R with S-R) [2]. Among these candidates, resistance random access memories (RRAMs) with self-rectifying characteristics in the low resistance state (LRS) has been receiving great attention in recent years owing to the feasibility of its realization in a simple one resistor structure. Typical bipolar resistive switching (RS) behavior of 1R with S-R cells, as indicated in Fig. 1b, reveals an asymmetric I–V characteristics in the LRS, which means that electrons can be easily injected or seriously blocked under different bias voltage. In this case, the ground scheme, where the selected word line (WL) is biased to V_read_ and all other WL/bit lines (BLs) are grounded, is generally adopted to mitigate leakage current (shown in lower right inset of Fig. 1b). The wealth of self-rectifying phenomena in different structures reported so far, while either too high switching voltage [3, 4] or insufficient ON/OFF window [5, 6] is exposed by most reports.

Presented in this work, therefore, is a material system that can provide ultralow operating current (<1 uA), sufficient ON/OFF window (~10^2^), median operating voltages (<6 V), as well as excellent self-rectifying functionality (RR >1000). In the bilayer stack, stoichiometric Ta_2_O_5_ and anoxic HfO_2-*x*_ were employed to be electrolytes, which contacted with high-work-function metal (Pt, 5.6 eV) and low-work-function metal (Hf, 3.9 eV), respectively [7]. The method can be generally stated as one layer (in this case, HfO_2-*x*_) and works as the RS layer by trapping and detrapping the deep trap sites while the other dielectric layer (in this case, Ta_2_O_5_) remains intact during the whole switching cycle and creates a high Schottky barrier with Pt to constitute the rectifying functionality.

## Methods

**Fig. 2:**
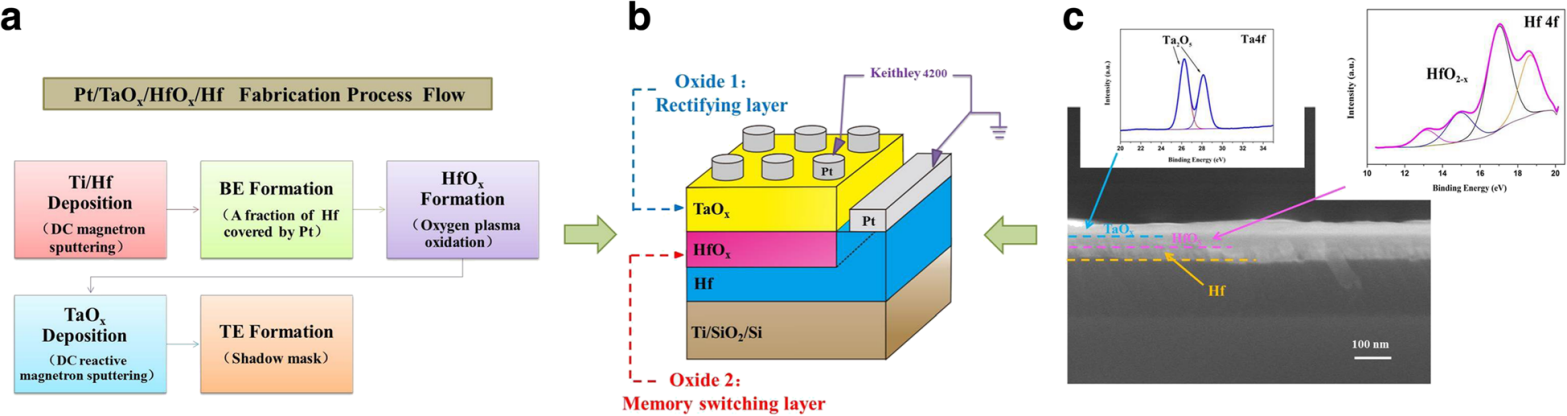
**a** Fabrication process flow for Pt/Ta_2_O_5_/HfO_2-*x*_/Hf stack. **b** Schematic of device layout and the electrical measurement configuration. **c** Typical FESEM cross sectional images of the Ta_2_O_5_(28 nm)/HfO_2-*x*_(oxidizing 1600 s)/Hf stack, along with its XPS data of each oxide layer (stoichiometric Ta_2_O_5_ and anoxic HfO_2_)

We fabricated the Pt/Ta_2_O_5_/HfO_2-*x*_/Hf devices by combining DC magnetron sputtering and oxygen plasma oxidation. The relevant fabrication process flow, equivalent schematic diagram, and cross sectional SEM image including relevant XPS data of our optimal sample are shown in Fig. 2. A bilayer of Ti and Hf, with a thickness of 3 and 60 nm, respectively, was deposited by DC magnetron sputtering on a SiO_2_/Si substrate. The HfO_2-*x*_ films were formed by directly oxidizing the metal Hf via oxygen plasma with the power of 50 W and different durations (i.e., 1300, 1600, and 1900 s). Whereas the 28-nm-thick Ta_2_O_5_ films were deposited by DC reactive magnetron sputtering using a Ta target in Ar/O_2_ gas mixture (with excess volume of O_2_ gas) at room temperature, the total pressure was ~0.28 Pa. For the electrode preparation, a large part of fresh metal Hf was reserved by covering aluminum foil in advance, and then the rest part was overlaid by inert Pt (30 nm) to fabricate the bottom-electrode Hf, while top-electrode Pt (50 nm) with a diameter of ~200 μm was deposited by DC magnetron sputtering using a metal shadow mask. For comparison, the stacks of Pt/HfO_2-*x*_/Hf without Ta_2_O_5_ layer and Pt/Ta_2_O_5_(28 nm)/Hf without HfO_2-*x*_ layer were also fabricated.

The cross sectional image was observed using a UKTRA-55 field emission scanning electron microscope (FESEM); and chemical status of films were examined using an X-ray photoelectron spectroscopy (XPS, Kratos Axis UltraDLD spectrometer, Kratos Analytical-A Shimadzu group company). For the XPS measurement, the Ar + ion beam energy was set to 1 keV during the sputter-etching. As to the electrical measurements, a low-noise Keithley 4200 semiconductor characterization system was conducted at room temperature, in voltage sweep mode. Each voltage sweep began from 0 V, and the bias was applied to the TE while the BE was grounded.

## Results and Discussion

**Fig. 3:**
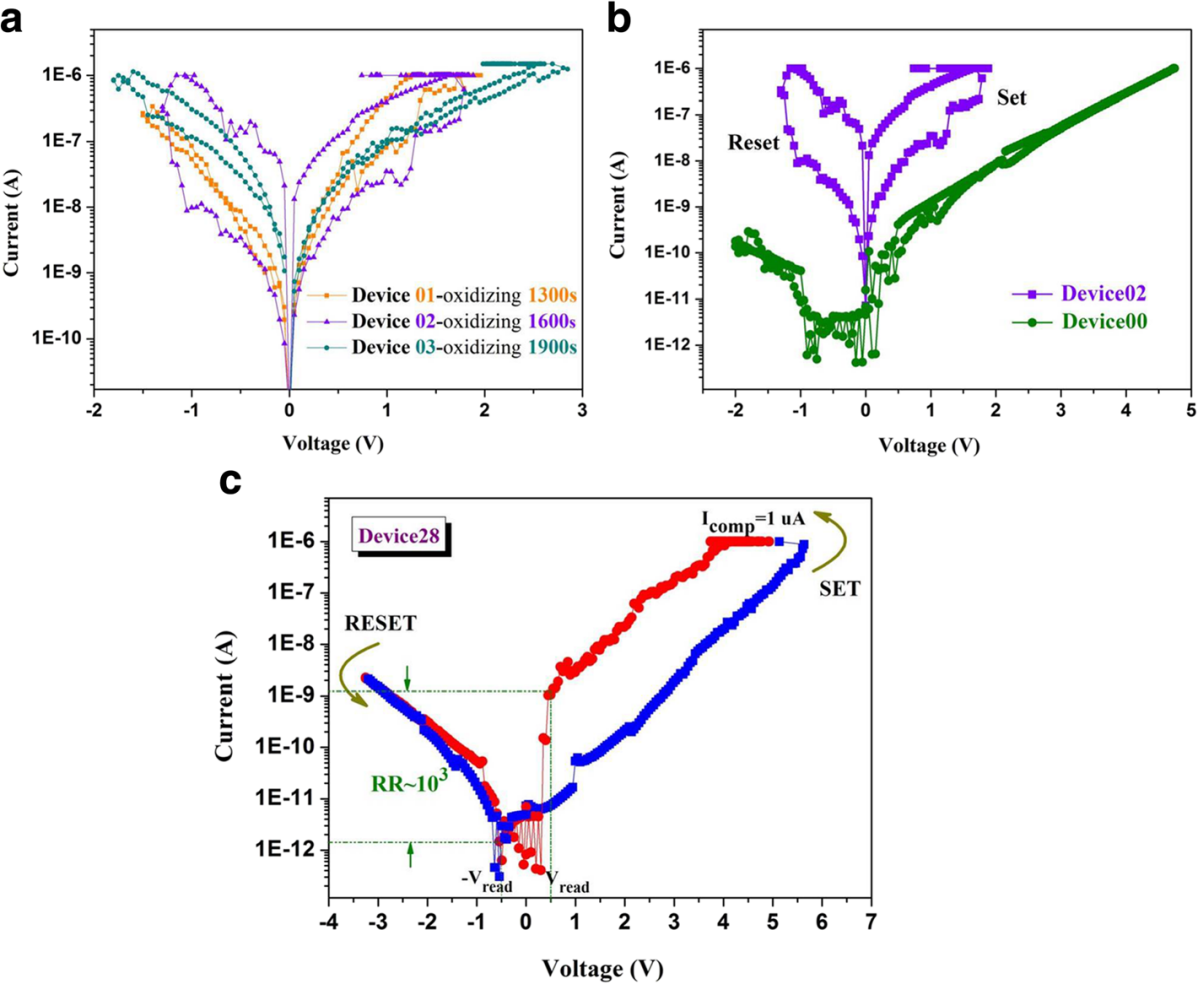
The semi-logarithmic I-V curves of **a** Device01-03 (*different oxidizing duration*), **b** Device02 (*violet curve*) and Device00 (*green curve*), and **c** Device28 with Pt/Ta_2_O_5_/HfO_2-*x*_/Hf structure

Figure 3 shows the typical I−V characteristics of our samples (deposited in the same batch). For simplicity, the device that possesses Pt/HfO_2-*x*_/Hf stack but different oxidizing duration (i.e., 1300, 1600, and 1900 s) is denoted as Device01, Device02, and Device03, respectively. Whereas Device00 and Device28 denote Pt/Ta_2_O_5_/Hf stack and the sample with Pt/Ta_2_O_5_(28 nm)/HfO_2-*x*_(oxidizing1600s)/Hf structure, respectively. As indicated in Fig. 3a, Device02 behaved better bidirectional RS characteristics than Device01 and Device03; therefore, this condition was chosen to prepare the RS layer in our Pt/Ta_2_O_5_/HfO_2-*x*_/Hf devices. Whereas rectifying properties (no RS behavior) was found in Device00, and the effect of their combination was reflected by Device28 (Fig. 3c). Additionally, it is worth noting from the XPS results in Fig. 2c that the binding energy of Ta 4f well coincided with the reported value of the Ta 4f peak (26.6 eV for Ta 4f7/2 and 28.4 eV for Ta 4f5/2) in Ta_2_O_5_ [3, 8]. For HfO_2-*x*_, grown by directly oxidizing the metal Hf via oxygen plasma with a duration 1600 s, the core-level Hf4f7/2–Hf4f5/2 was observed at an energy corresponding to the non-stoichiometric HfO_2_ (12.7–18.5 eV) [9], which indicated the presence of the neutral oxygen vacancy in our HfO_2-*x*_ layer. Along with the I–V characteristics in Fig. 3, it was confirmed that HfO_2-*x*_ layer plays the role of RS layer indeed, whereas stoichiometric Ta_2_O_5_ layer works as a rectifier with high resistance and only slight oxygen deficiency. Beyond that, the thickness of HfO_2-*x*_ layer with oxidizing duration 1600 s approximates 30 nm.

**Fig. 4:**
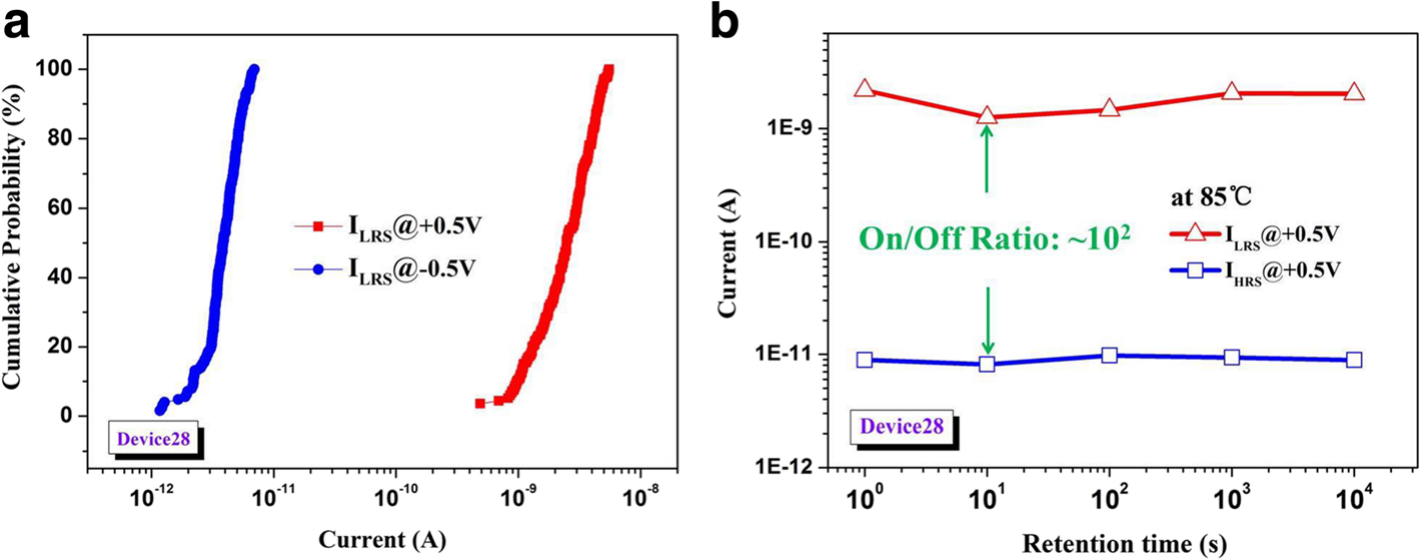
**a** Distribution of the I_LRS_ (at ±0.5 V) under DC sweeping mode for Device28 and **b** its retention property

When a positive voltage sweep with an *I*_comp_ of 1 uA (to prevent the device hard breakdown) were firstly applied whereas their BE were grounded, then as-fabricated devices transform into the LRS (or ON state), i.e., the SET process (for Device02 and Device28). Particularly the electroforming was relieved since a large amount of oxygen vacancies (*V*_*O*_) in the HfO_2-*x*_ film (RS layer) [10, 11]. To turn the devices back into the HRS, i.e., the RESET process, a negative voltage sweep (−1.5 V for Device02 and −3.5 V for Device28) was required. Particularly, after stacking a 28-nm-thick Ta_2_O_5_ layer on HfO_2-*x*_ film, a pronounced self-rectifying behavior appeared in Device28. Significantly, a ~4 × 10^3^-fold reduction in LRS current was observed at −0.5 V(−V_read_) compared with the current at 0.5 V(V_read_). In addition, a DC cycling and retention property test of Device28 were also implemented, the results are presented in Fig. 4. The high uniformity of RS performance and stable distribution of retention time data (baked at 85 °C up to 10^4^ s) indicate the high feasibility of the present structure as the RS element in crossbar.

**Fig. 5:**
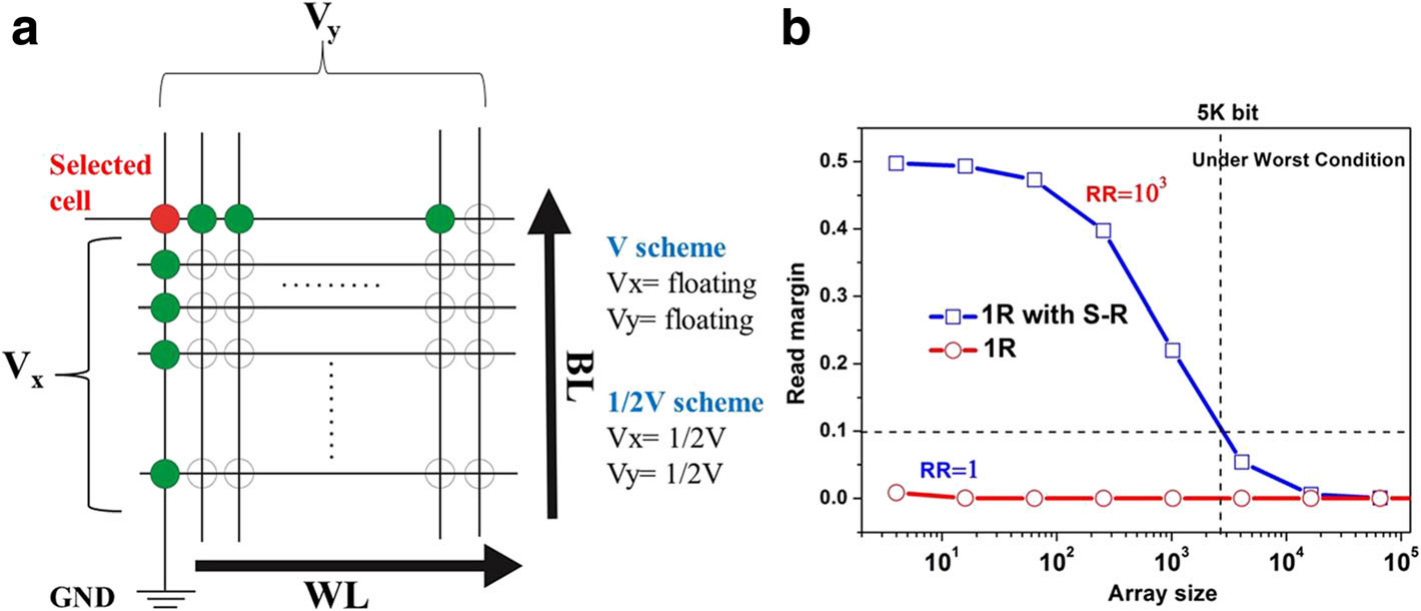
**a** Schematic of data reading (V, V/2) and **b** comparison of calculated readout margins in *N × N* crossbar array integrating RRAM cells with and without self-rectifying behavior

To demonstrate the feasibility of settling the sneak-path issue by utilizing the observed self-rectifying behavior, numerical simulation of the maximum crossbar size has also been conducted for Device28 by the use of MATLAB. Figure 5 shows that the obtained self-rectifying property in this work can guarantee a maximum crossbar array size of ∼5 k bit in the worst case scenario for reading (V scheme).

**Fig. 6:**
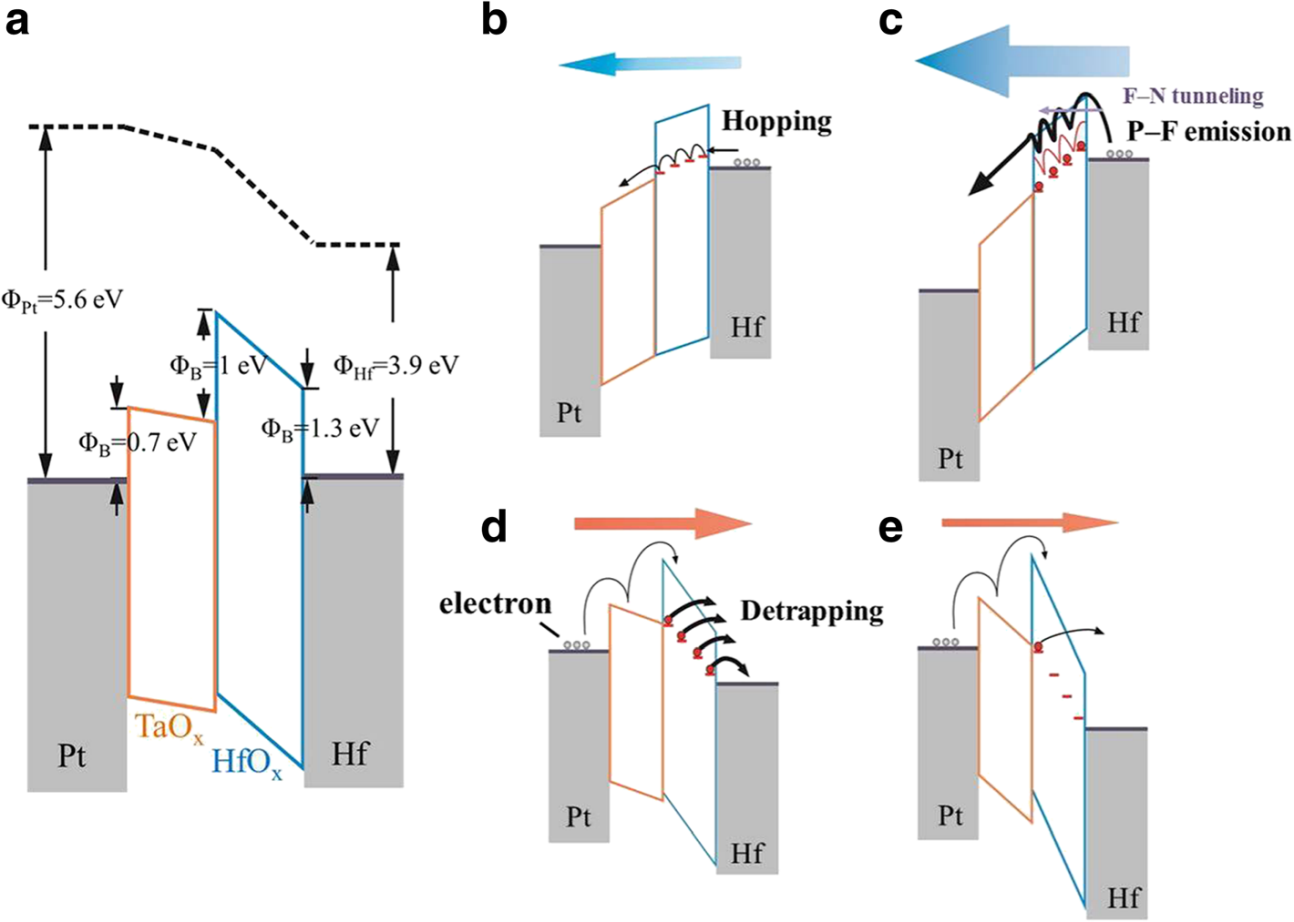
Schematic diagram showing the switching and self-rectifying mechanism of our Devices. **a** Schematic diagrams of band structure for zero bias condition. **b**–**e** The schematic band diagram under different voltage bias

Now, we turn to discuss the switching and self-rectifying mechanism discovered in Device28 from the viewpoint of energy band. Although the accurate energy band structure could not be depicted due to the lack of precise information on the band offset between the two dielectric layers and possible (larger) Schottky barrier at the HfO_2-*x*_/Hf interface, a tentative schematic band diagram could be drawn as shown in Fig. 6a for the zero external bias condition. Here, the CB offset between Ta_2_O_5_ and HfO_2-*x*_ was taken as 1 eV considering the known electron affinities of both materials [12, 13], and contact potential at the HfO_2-*x*_/Hf interface was taken as 1.3 eV based on their electron affinity and work function. As the band gap of Ta_2_O_5_ is lower than that of HfO_2-*x*_ (≈4.2 and ≈5.68 eV, respectively) [3], the electrons transported to Ta_2_O_5_ easily moved to Pt TE via the conduction within the CB of the Ta_2_O_5_ layer, so that the Ta_2_O_5_ layer kept intact under the positive bias condition. In addition, considering the high oxygen affinity of beneath metal Hf in the HfO_2-*x*_/Hf stack, abundance of *V*_*O*_ would be induced in bulk portion of HfO_2-*x*._ It is believed that these *V*_*O*_ values were dispersed within the HfO_2-*x*_ layer and acted as the deep trap sites (red dot lines drawn in Fig. 6b–e) for the injected carriers in both states [3]. (It must be noted that the trap depth of 1.0 eV well coincided with the energy distance between the *V*_*o*_ and the CB edge in HfO_2-*x*_ [14]. Therefore, the trap positions of HfO_2-*x*_ layer occur near E_c_ of Ta_2_O_5_ layer.)

When a small positive bias was applied to the Pt TE, a small amount of injected electrons, which tunneling from cathode to traps, were interfered with by the deep trap and transported through the HfO_2-*x*_ via hopping mechanism, so that the current flow under this circumstance must be much lower, as schematically shown in Fig. 6b. The observation of initially low current in Fig. 3b suggests that the deep trap levels were with the trap-empty configurations under low positive bias condition, which well coincided with the HRS. As the positive voltage increased, the carrier injection became higher and the energy band of electrolytes would be tilted further, so that traps started to be filled with major injected carriers, and the others would tunnel from Hf BE to the E_c_ of HfO_2-*x*_ layer (F-N tunneling). Actually, the subsequent emission from traps to the E_c_ of HfO_2-*x*_ layer is essentially the Poole–Frenkel emission [15]. At the same time, the whole system switched to LRS (as indicated in Figs. 3b and 6c). The switching back from the LRS to HRS under the negative bias could be understood as follows.

After withdrawing the positive bias and applying a negative bias to the Pt TE, the electrons in the HfO_2-*x*_ traps started to detrap continually while the electron injection from the Pt TE was suppressed by the high Schottky barrier height, Fig. 6c shows the schematic diagram of this circumstance. When the traps became empty by the high negative bias, and bias was removed subsequently, the energy band diagram could be represented by Fig. 6e.

The most critical feature of the abovementioned switching mechanism is the change in the charge state of the electron traps, presumably *V*_*O*_ with different oxidation states in HfO_2-*x*_, not the variations in their local spatial distribution or concentration. Note that it is hopeful that improve the rectifying properties of our device if its active area can scaling further.

## Conclusions

In summary, the RRAM device with ultralow operating current (<1 uA), sufficient ON/OFF ratio (~10^2^), median operating voltages (<6 V), as well as excellent self-rectifying properties was prepared in a simple Pt/Ta_2_O_5_/HfO_2-*x*_/Hf structure successfully. And satisfactory switching uniformity and retention performance are also demonstrated in it. In the stack, Ta_2_O_5_ layer works as a rectifier with high resistance and only slight oxygen deficiency, while HfO_2-*x*_ layer plays the role of the RS layer with more oxygen deficiency, lower dielectric constant, and higher energy band gap. These abovementioned merits manifest that the prototype Pt/Ta_2_O_5_/HfO_2-*x*_/Hf devices could be used to effectively mitigate the sneak leakage in crossbar RRAM arrays.
